# Generation of femtosecond polygonal optical vortices from a mode-locked quasi-frequency-degenerate laser

**DOI:** 10.1038/s41377-025-01902-1

**Published:** 2025-06-23

**Authors:** Hongyu Liu, Lisong Yan, Liang Wang, Dongfang Li, Shenao Zhang, Xin Liu, Heyan Liu, Kunjian Dai, Qing Wang, Jinwei Zhang

**Affiliations:** 1 School of Optical and Electronic Information and Wuhan National Laboratory for Optoelectronics, Huazhong University of Science and Technology, Wuhan, China; 2 School of Optics and Photonics, Beijing Institute of Technology, Beijing, China

**Keywords:** Ultrafast lasers, Mode-locked lasers, Solid-state lasers

## Abstract

Polygonal optical vortices—a new subset of optical vortices—uniquely enable numerous applications due to the new degrees of freedom offered and their customizable light intensity structure. So far, their generation has only been reported for continuous wave. Here, we demonstrate the first femtosecond polygonal optical vortex pulses, which is also the first pulsed demonstration of such vortices in general. From a mode-locked Yb:KGW laser oscillator, femtosecond Hermit-Gaussian pulses at the quasi-frequency-degenerate state were generated and subsequently converted by astigmatic mode conversion to femtosecond polygonal optical vortices. They have light intensity distributions of square, pentagonal, and hexagonal shapes and carry orbital angular momentums. In all these variations, the average power and pulse duration are greater than one watt and less than 500 fs. These results open the way to new applications in the fields of femtosecond optical tweezer and three-dimensional microstructure fabrication.

## Abstract (web summary)

Femtosecond polygonal optical vortices with light intensity distributions of square, pentagonal, and hexagonal shapes were generated from a passive mode-locked solid-state laser oscillator.

## Graphical abstract

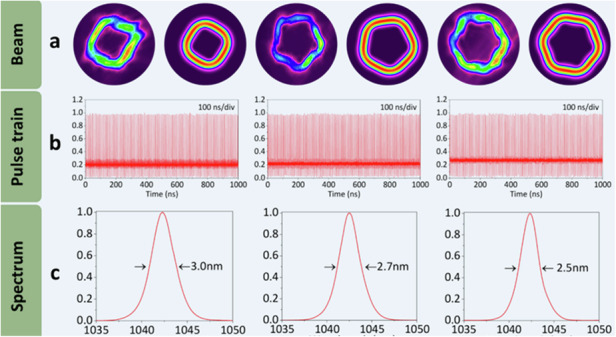

## Introduction

Vortex beams, having unique singularity characteristics in the wavefront and spiral phase distribution, carry orbital angular momentum (OAM) and are used in a wide variety of applications such as optical communication^1–4^, trapping and guiding of micro-particles^5,6^, sub-diffraction limit microscopy^7^ and control of multi-dimensional states in quantum entanglement^8–11^. In contrast to continuous-wave (CW) vortex beam^12^, femtosecond optical vortex (FOV) pulses not only have helical phase wavefront in the transverse mode but also exhibit high peak power. As such, they are attractive for many novel applications, including the generation of attosecond vortices through high-order harmonic generation^13^, particles manipulation using FOVs^14,15^, exploring the dynamics of the interaction between matter and complex light fields^16^ and fabrication of three-dimensional (3D) chiral microstructures^17^.

To date, substantial progress has been made to improve FOVs maximum tunable order^18,19^, shorten their pulse duration^20,21^, control their phase singularity^22^ and expand their wavelength range^23,24^. These advances have greatly promoted the potential applications of FOVs. Meanwhile, research efforts are also implemented, aiming at adding new degrees of freedom to FOVs. In recent years, a new type of polygonal optical vortex (POV) beam has attracted widespread attention. Unlike traditional circular vortex beams, POV refers to vortex beams carrying OAM with a closed polygonal pattern in intensity distribution, offering a new degree of freedom on the shape of the beam profile. Several methods have been proposed to generate POV through the transformation of a Gaussian laser beam, such as using the optical pen^25^, all-dielectric geometric metasurface^26^, free lens modulation^27^, and high-order cross-phase at the plane of a spatial light modulator^28,29^. In addition, POV beam was recently generated based on the Hermite-Gaussian (HG), oscillator followed by an astigmatic mode converter (AMC)^30^, which can reduce the cost and complexity of the setup while enhancing the robustness of the generation. These methods are used to generate POV with arbitrary polygonal structures, and the switching between different polygonal structures has also been demonstrated. Nevertheless, the generated POVs are still limited to the CW regime, and the generation of ultrafast POVs has not been reported yet.

Extending POVs to the femtosecond regime—forming femtosecond polygonal optical vortices (FPOVs)—will endow it with extremely short pulse duration and high peak power, opening up many potential applications. The FPOV pulses can stimulate nonlinear absorption dynamics such as multi-photon absorption that permits not only surface processing, but also 3D internal microfabrication of transparent materials^31^. When combined with the OAM characteristics and polygonal intensity structure, these pulses uniquely enable the fabrication of complex 3D spiral microtubes with controllable geometry parameters^32^. Furthermore, a FPOV source can be utilized as femtosecond optical tweezers^33^. With the extremely high intensity of the femtosecond beam, the local refractive index of the trapped particles could be changed nonlinearly, which allows additional degrees of freedom for their capture and control. Compared with traditional center symmetric circular FOVs, FPOVs with different intensity structures can be used to capture particles of different shapes, thereby sorting particles of different shapes and achieving higher particle screening efficiency^34^.

One possible approach to generate FPOVs is to employ phase modulation devices to shape a femtosecond laser beam. However, this is often hindered by strong spatial dispersion^35^, phase singularity splitting^36^, low damage threshold, and low conversion efficiencies, which restrict the power scaling capacity of the FPOV as well as the compression of pulse duration. Compared with this method, a mode-locked HG oscillator with an AMC is more promising for generating high-power FPOVs with high stability. The use of AMC has been verified to be an effective way for converting the HG modes to Laguerre-Gaussian (LG) modes^37^ and is widely applied in the generation of FOVs because of its high damage threshold, wide operating range, and high mode purity. As reported in the CW regime, the HG oscillator needs to be operated at quasi-frequency-degenerate (QFD) modes^30^, which consist of multiple phase-locked HG modes under an off-axis pumping condition. To generate FPOV, the longitudinal mode locking is additionally required while the transverse modes satisfy the QFD situation, which is the key challenge that we need to address.

In this work, a sophisticated solid-state Yb:KGW oscillator was built, and the longitudinal laser modes were passively locked with a semiconductor saturable absorber mirror (SESAM). Femtosecond HG pulses in the QFD states were delivered from the oscillator and subsequently converted to FPOVs with the AMC. By adjusting the cavity structure to meet different QFD states, we obtained FPOVs with square, pentagonal, and hexagonal intensity distributions. The pulses all have average powers beyond one watt, and the pulse durations are <500 fs. This is the first demonstration of FPOVs, which is also the first pulsed POVs in general, to the best of our knowledge.

## Results

### Experimental setup

**Fig. 1:**
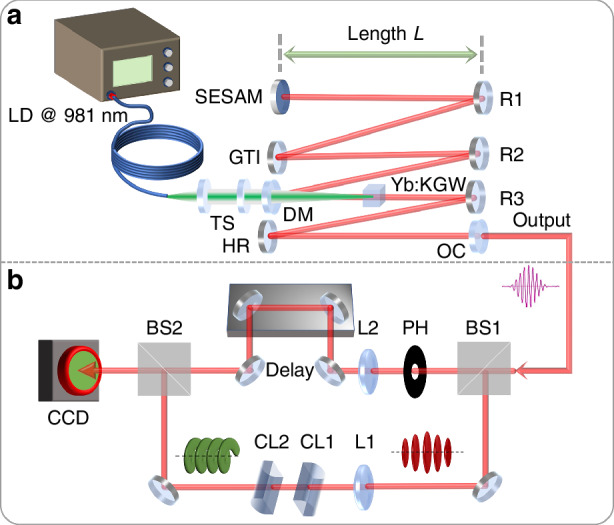
Schematic of the experimental setup. **a** Oscillator setup. LD laser diode, TS telescope system, DM dichroic mirror, HR high-reflectivity mirror, R1, R2, and R3: concave mirrors with a radius of curvature of −150 mm, −200 mm and −200 mm, respectively, OC output coupler with a transmission of 3%. **b** Schematic of a home-built Mach-Zehnder interferometer and AMC. PH pinhole, L1, L2 lenses with a focal length of 200 mm and 150 mm, respectively, CL cylindrical lenses, BS beam splitter

The schematic of the experimental setup is depicted in Fig. 1. A Yb:KGW oscillator was built to generate the initial pulses operating at a repetition rate of ~116 MHz. The pump beam at 981 nm was generated from a fiber-coupled laser diode and focused into the laser crystal by a telescope system with a focused beam diameter of 105 μm. The fiber end and the telescope system were placed on a 3D translation stage so that the pump beam could be shifted relative to the laser beam axis. A standing-wave cavity was designed with a beam waist diameter of ~100 μm for the fundamental laser mode inside the crystal. The round-trip group delay dispersion was −4000 fs^2^, provided by a GTI (Gires-Tournois interferometer) mirror. The SESAM was used as an end mirror for longitudinal mode locking and placed on the translation stage so that we could move it and adjust the distance *L* between the SESAM mirror and a concave mirror R1. To achieve a high-power density on the SESAM and initiate passive mode locking, R1 has a radius of curvature of −150 mm. The beam diameter on the SESAM was designed to be 80 μm for the fundamental transverse mode. A home-made Mach-Zehnder interferometer (shown in Fig. 1b) was built to analyze the phase singularity and the topological charge number of the generated FPOVs. In one arm of the Mach-Zehnder interferometer, we built an AMC system consisting of two cylindrical lenses for mode conversion.

### Generation of POV in CW regime with tunable polygonal structures

**Fig. 2:**
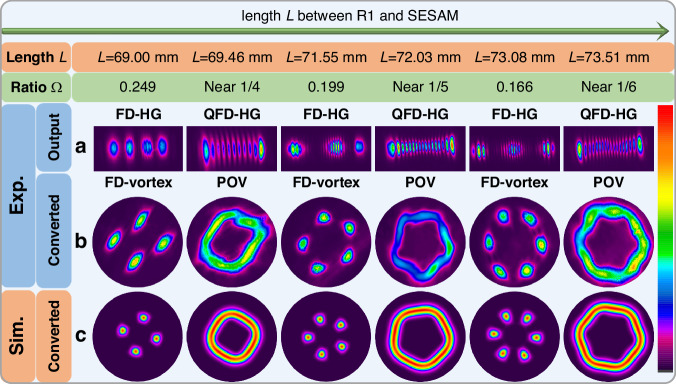
**Beam patterns of the laser beam in CW regime at different distances L and mode-spacing ratio Ω.** **a** Beam patterns of FD-HG modes and QFD-HG modes generated from the oscillator cavity. **b** Beam patterns of FD vortex modes and POV modes after the conversion by the AMC. **c** Simulated beam patterns of FD vortex modes and POV modes after the conversion by the AMC

The mode-spacing ratio of a laser oscillator is defined as Ω *=* Δ*f*_*T*_*/*Δ*f*_*L*_^38^, which is a ratio between the longitudinal mode spacing *Δf*_*L*_ = c*/(*2 L*)* and the transverse mode spacing Δ*f*_*T*_ = Δ*f*_*L*_*θ*_G_(L)/π, with *θ*_G_(z) = tan^−1^(z/z_R_) being the Gouy phase. It can be changed by accurately adjusting the distance *L* between the SESAM and R1 as a result of the modification of the one-way transfer matrix of the laser cavity. Initially, we implemented the translation-based off-axis pumping scheme in the oscillator cavity to generate a pure HG_13,0_ mode in the CW regime^39,40^. Since only a single HG mode was stimulated in the oscillator, the mode-spacing ratio Ω was an irrational number. In this case, the laser oscillator operated in a non-frequency-degenerate state. Next, we tuned the distance *L*, and the cavity was then operated in the FD condition where the ratio Ω became a rational number (see the first part of Materials and methods). During the experiment, the mode-spacing ratio Ω was adjusted to unit fractions from 1/4, 1/5, to 1/6 with increasing *L*, and the corresponding frequency-degenerate Hermite-Gaussian (FD-HG) modes were emitted from the laser cavity. Based on the FD state, a further slight adjustment of the distance *L* will enable the oscillator to change to the QFD state that consists of multiple phase-locked HG modes (quasi-FD-HG modes, QFD-HG modes). We measured the intensity profiles of the HG beams from the oscillator under both FD and QFD states, as shown in Fig. 2a. After passing through the AMC, the laser beams were converted into FD vortex modes with multi-point pattern and POV modes, respectively (see Fig. 2b). The principle of mode conversion by an AMC is described (see the third part of Materials and methods), and the simulated transition process from QFD-HG mode to POVs is shown in Fig. S1 (Supplementary Information).

Specifically, the initial distance *L* was set at 69 mm, and the oscillator was operated under an FD state with Ω = 1/4, which generated FD vortex mode exhibiting four bright isolated spots after the conversion from AMC. The calculated mode-spacing ratio is Ω = 0.249, matching well with the experimental behavior. The oscillator then changed to the QFD state when we slightly increased the distance *L* to 69.46 mm. POV mode with a square light intensity distribution was obtained after the AMC conversion. Further increase of the length *L* to 71.55 mm and 73.08 mm resulted in the FD state of Ω = 1/5 and Ω = 1/6, and the calculated mode-spacing ratio was Ω = 0.199 and Ω = 0.166, respectively. The converted FD vortex mode patterns for these two states have, respectively, five and six isolated spots. Similar to the situation of Ω = 1/4, QFD states near the FD state of Ω = 1/5 and Ω = 1/6 were obtained by slightly increasing the distance *L* to 72.03 mm and 73.51 mm, respectively. At these two states, we realized the generation of POV modes with pentagonal and hexagonal intensity distributions. Figure 2c shows the simulated results of the converted modes, which are in good agreement with the experimental results.

### Generation of FPOV pulses with square, pentagonal and hexagonal patterns

For the generation of the FPOV pulses, a SESAM was adopted to initiate and maintain the passive locking of the longitudinal modes. Compared to the fundamental transverse modes, high-order transverse modes had larger beam sizes and higher diffraction losses, which often led to low power density and Q-switched pulses. In order to realize a stable longitudinal mode locking, the oscillator cavity was designed to have a small spot size at the SESAM, and the pump power needed to be increased to a certain level. At the QFD state near Ω = 1/4, the longitudinal mode locking was realized when the pump power reached 17.5 W, and FPOV pulses with square pattern were obtained behind the AMC with an average power of 1.3 W. Similarly, when the oscillator was operated at QFD states near Ω = 1/5 and Ω = 1/6, FPOV pulses with pentagonal and hexagonal patterns were achieved under a pump power of 17.6 W and 18.6 W, respectively. The average power was 1.8 W for pentagonal FPOV pulses, and 1.2 W for hexagonal FPOV pulses. Under these three operating conditions, the average powers of the femtosecond QFD-HG pulses delivered directly from the laser cavity were 1.4 W, 1.9 W and 1.3 W, corresponding to optical-to-optical efficiencies of 8%, 10.8% and 7%, respectively. The subsequent transformation of these femtosecond QFD-HG pulses into FPOV pulses exhibited a conversion efficiency of ~90% due to the coating-induced losses in the AMC stage. Compared with conventional high-order femtosecond LG vortices with identical topological charges, the FPOV pulses demonstrate an expanded gain area resulting from their multi-transverse-mode composition, which consequently enables higher optical-to-optical efficiency^41^.

**Fig. 3:**
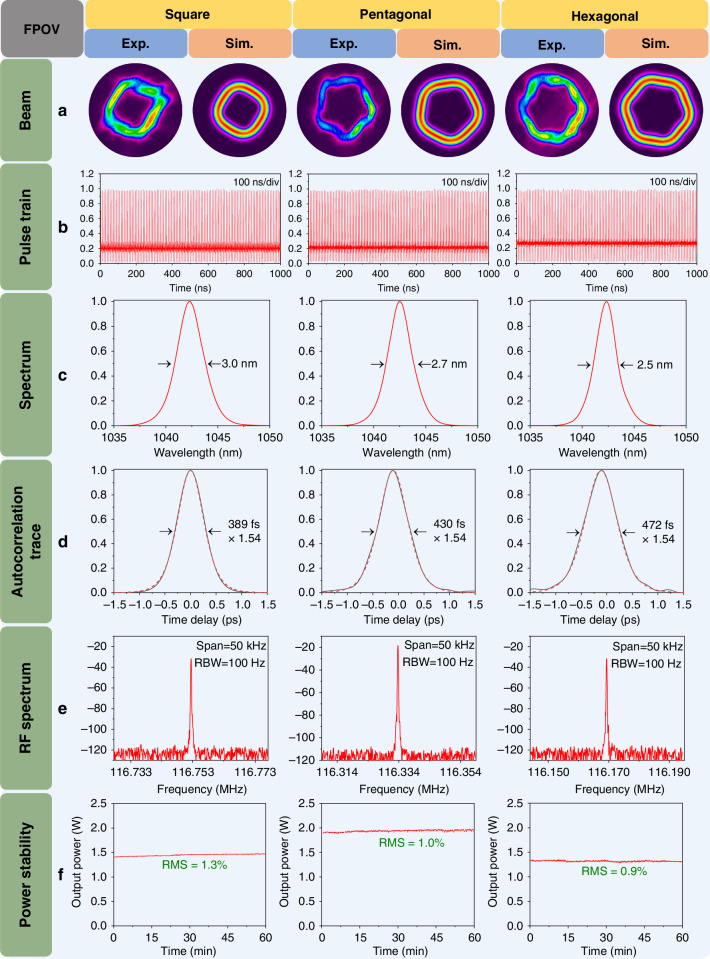
**Characteristics of the generated square, pentagonal and hexagonal FPOV pulses.** **a** Measured and simulated beam patterns of square, pentagonal and hexagonal FPOV pulses, respectively. **b** Pulse trains on the oscilloscope with a time scale of 100 ns/div. **c** Measured spectra of FPOV pulses; **d** Autocorrelation traces; **e** RF spectra of FPOV pulses. **f** Average power stability measurement of FPOV pulses within one hour, showing a deviation of 1.3%, 1.0%, and 0.9% (RMS), respectively

The beam patterns of FPOV pulses were measured and shown in Fig. 3a, which exhibited the various polygonal structures, including square, pentagonal and hexagonal. Figure 3a also shows the simulated beam patterns of the three FPOVs, which agree well with the experimental results. By performing a quantitative normalized cross-correlation function between the normalized experimental and simulated patterns^42^, we determined the mode purities of the three FPOVs to be 95.5%, 96.2% and 96.1%, respectively. The high mode purities indicate negligible mixing of other undesired higher-order modes in the generated FPOVs. The measured pulse trains of the three FPOV pulses on the oscilloscope are shown in Fig. 3b with a time scale of 100 ns/div, indicating continuous longitudinal mode locking without Q-switching behavior. The spectra and autocorrelation traces of the FPOV pulses under the three states are shown in Fig. 3c, d, revealing pulse durations below 500 fs (assuming a sech^2^ fit) in all cases. Figure 3e shows the radio frequency (RF) spectra with high signal-to-noise ratios of ~80 dB, all measured at a resolution bandwidth (RBW) of 100 Hz. It is worth noting that there are slight differences in the repetition frequency among the different FPOV pulses, which are caused by the change in the distance *L*. No additional beat signals were detected in the RF spectra, indicating both the absence of spurious mode components and stable mode locking in all these situations. To further verify the stability of the QFD state and FPOV pulses, the average power stability of three FPOVs was monitored within 1 hour (see Fig. 3f). The measured power deviations were ~1.3%, 1.0%, and 0.9% (root mean square, RMS), respectively, demonstrating excellent long-term operational stability of both the QFD state and the FPOVs even though the laser system was built directly on the optical table without an enclosed housing. Additionally, the beam profiles of the FPOVs were recorded at ten-minute intervals for one hour (see Fig. S2, Supplementary Information). The results showed that both the polygonal structures and intensity distributions remained highly stable throughout continuous operation, further validating the exceptional beam stability of the demonstrated FPOVs. All the parameters of the laser beams are summarized in Table 1.

**Table 1:** Summary of the parameters for the three types of FPOV pulses

FPOV	*P*_Pump_ (W)	*P*_QFD-HG_ (W)	*P*_FPOV_ (W)	*τ*_s_ (nm)	*τ*_p_ (fs)	*f*_*rep*_ (MHz)	*η*_*O-O*_	*β*	*S*_*p*_
Square	17.5	1.4	1.3	3.0	389	116.8	8.0%	95.5%	1.3%
Pentagonal	17.6	1.9	1.8	2.7	430	116.3	10.8%	96.2%	1.0%
Hexagonal	18.6	1.3	1.2	2.5	472	116.2	7.0%	96.1%	0.9%

*P*_Pump_ pump power, *P*_QFD-HG_ average power of the QFD-HG pulses, *P*_FPOV_ average power of the FPOV pulses after the conversion from QFD-HG modes, *τ*_s_ spectra width of the FPOV pulses, *τ*_p_ pulse width of the FPOV pulses, *f*_*rep*_ repetition frequency of the FPOV pulses, *η*_*O-O*_ optical-to-optical efficiency from the pump laser to the generated femtosecond QFD-HG laser, *β* mode purity calculated by performing the quantitative normalized cross-correlation function between the experimental FPOVs and the simulation FPOVs, *S*_*p*_ the measured power stability of femtosecond QFD-HG laser (RMS)

In order to analyze the phase singularity and the topological charge number of the FPOVs, we built a Mach-Zehnder interferometer (shown in Fig. 1b) to inspect the interference pattern between the FPOV beam and a spherical wave, thereby characterizing the spatial phase properties. The interferometer included an optical delay line following a pinhole. A part of the QFD beam was selected and collimated afterwards to a coherent near plane wave by a lens (L2), which acted as the reference beam to be interfered with the converted FPOV beam. The AMC system was contained in the other arm to deliver FPOV pulses carrying OAMs. By matching the time delay between the FPOV pulses and the reference pulses and overlapping them spatially on the CCD camera, clear interference fringes became visible.

**Fig. 4:**
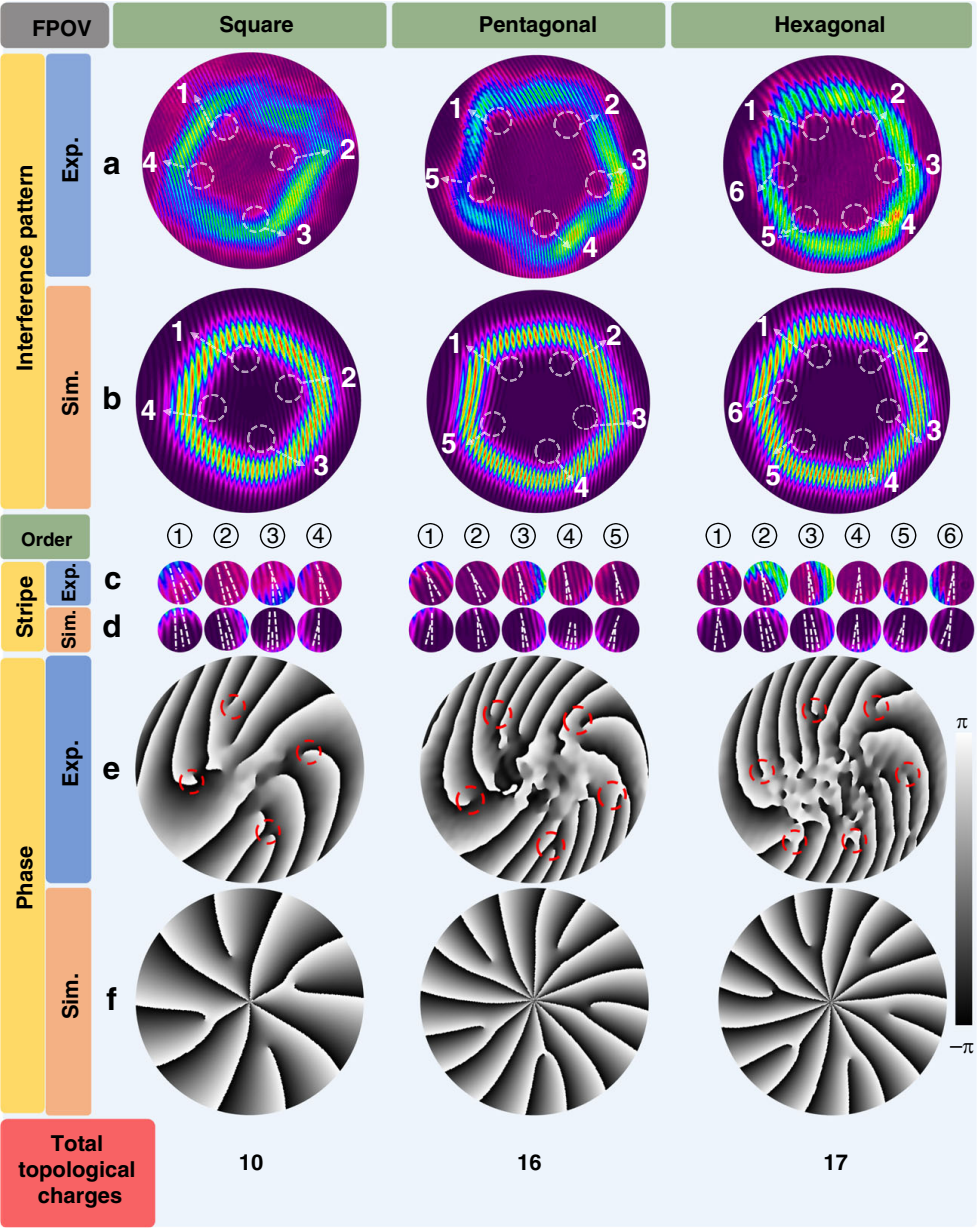
**The phase singularity and topological charge characteristics of different FPOVs.** **a**, **b** Measured (**a**) and simulated (**b**) interference patterns of square, pentagonal and hexagonal FPOV pulses, with white dashed circles highlighting regions of interest where fork-like interference patterns reveal singularities and their associated topological charges. **c**, **d** Magnified views of the highlighted regions in (**a**) and (**b**), showing the detailed interference fringes in the experimental (**c**) and simulated (**d**) results, respectively. **e**, **f** Experimental (**e**) and simulated (**f**) phase distributions of the three FPOVs, illustrating the spatial arrangement of singularities and the chirality of the topological charges. The total number of topological charges for each FPOV is labeled below the corresponding phase maps

The phase characteristics of the FPOV are further analyzed through observations of the interference stripes, enabling precise mapping of singularities and topological charges. Figure 4a, b illustrates the experimental and simulated interference patterns of the three FPOV pulses. The fork-like fringe patterns observed in these images, caused by singularities and topological charges, allow for an accurate determination of their positions and quantities, as highlighted by the white dashed circles. By calculating the difference in the stripe number between the upper and lower regions of the interference patterns, the total topological charges of the three FPOVs are obtained as 10, 16, and 17, respectively. It is worth noting that singularities with high-order topological charges exist at the beam centers but are not visible due to the absence of intensity in the central regions. Interestingly, the number of the highlighted regions matches the number of polygonal sides for each FPOV, with these regions located at the internal corners of the patterns. To observe the detailed features, magnified views of these regions are displayed in Fig. 4c, d for the experimental and simulated results, respectively. The dashed lines represent the positions of the interference fringes, while the fork-like shapes confirm the existence of a topological charge of one in these regions. A strong agreement between the experimental and simulated results is observed. For a more comprehensive description of the FPOV characteristics, Fig. 4e, f illustrates the experimental and simulated phase patterns of the three FPOVs. The experimental phase maps are recovered from the interference patterns measured in Fig. 4a^43^. The phase singularities located at the internal corners are delineated by red dashed circles, while the ambiguous phase distribution observed at the center of each phase map arises from the negligible light intensity in the central region of the FPOV. Through quantitative analysis of the spiral phase branches present in the experimental phase maps, the total topological charges of the three FPOVs were determined. These values exhibit excellent agreement with the topological charges obtained via interference fringe counting in the corresponding interference patterns, thereby validating the accuracy of our experimentally reconstructed phase profiles. These phase maps offer an intuitive representation of the distributions of the singularities and the topological charges across the beam profiles. The phase variation from -π to π around a singularity signifies a single topological charge, and the topological charges in each FPOV exhibit identical chirality. Specifically, the square FPOV contains 6 topological charges at the beam center and 4 at the corners of the beam pattern. For the pentagonal and hexagonal FPOVs, the beam centers host 11 topological charges, while 5 and 6 charges are distributed at the corners, respectively. The total topological charges for each FPOV are labeled at the bottom of Fig. 4, providing a clear quantitative summary of their characteristics.

To verify the stability of the topological charge over time, we monitored the phase distribution of the square, pentagonal and hexagonal FPOVs pulses within one hour (see Fig. S2, Supplementary Information). The results demonstrated high stability in both the phase structure and topological charge. However, these measurements were time-averaged and thus did not resolve the variations between different pulses. In order to investigate this, the FPOVs pulses were selected by high-speed Pockels cells, reducing the repetition frequency to 50 kHz, 100 kHz and 1 MHz, respectively. The measured interference patterns of these selected pulse trains (see Fig. S3, Supplementary Information) consistently exhibited stable total topological charges of 10, 16, and 17 for square, pentagonal, and hexagonal FPOVs, respectively, confirming the robustness of their OAM characteristics over time. For the next step, pulse-to-pulse stability of OAM will be examined through pulse-resolved interferometry.

**Fig. 5:**
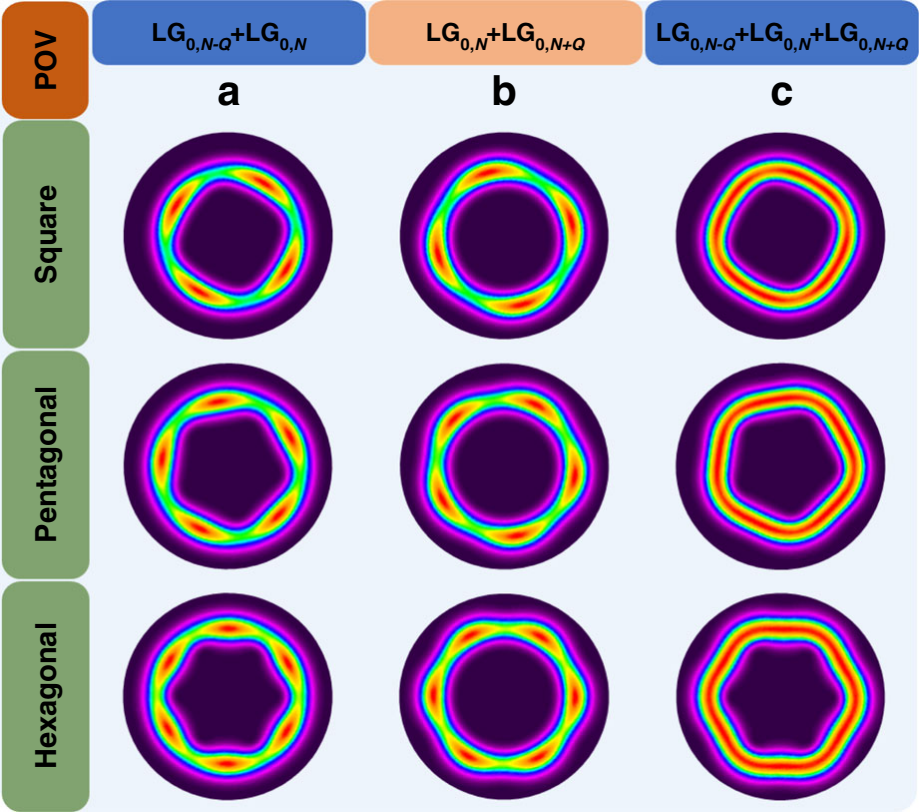
**The simulated intensity patterns of transverse mode locking with different LG modes.** Simulation parameters: the order of the primary mode *N* = 15, the phase factor *ϕ*_0_ = π/2, *c*_0_ = 0.15, *P* = 1, *Q* = 4, 5 and 6, corresponding to the square, pentagonal and hexagonal POVs, respectively. **a** ${\psi }_{0,N,S}^{\text{LG}}+{c}_{0}{\psi }_{0,N-Q,S+1}^{\text{LG}}\exp (-i{\phi }_{0})$ exhibits the polygonal structure of inner edges; **b** ${\psi }_{0,N,S}^{\text{LG}}-{c}_{0}{\psi }_{0,N+Q,S-1}^{\text{LG}}\exp (i{\phi }_{0})$ exhibits the polygonal structure of outer edges; **c** ${c}_{0}{\psi }_{0,N-Q,S+1}^{\text{LG}}\exp (-i{\phi }_{0})+{\psi }_{0,N,S}^{\text{LG}}-{c}_{0}{\psi }_{0,N+Q,S-1}^{\text{LG}}\exp (i{\phi }_{0})$ represents POV patterns with the complete polygonal structure

We present a mathematical formulation that describes the generation mechanism of POV beams (see Eq. (8) in the second part of Materials and Methods). The theoretical formulation reveals that the POVs are formed by the coherent superposition of three types of LG modes, which include one primary mode (LG_0,*N*_) and two secondary modes (LG_0,*N-Q*_ and LG_0,*N+Q*_). Analysis on the eigenmode frequency of the laser cavity demonstrates that these three LG modes share identical eigenmode frequency, a consequence of the QFD cavity design (see the first part of Materials and Methods). This frequency degeneracy ensures that POVs generated from these mode combinations will produce stable pulse trains devoid of beat signals when longitudinal mode-locking is achieved. To clarify the transverse mode interaction dynamics, Fig. 5 presents a numerical simulation of transverse mode-locking configurations, first between the primary LG_0,*N*_ mode and each secondary mode individually, and then with all three modes locked simultaneously. When phase-locking occurs between the primary LG_0,*N*_ mode and the lower-order secondary LG_0,*N-Q*_ mode, their coherent superposition with a fixed phase difference generates an output field exhibiting a stable polygonal structure with well-defined inner edges (Fig. 5a). Similarly, mode-locking of the primary LG_0,*N*_ mode with the higher-order secondary LG_0,*N+Q*_ mode produces a complementary polygonal structure featuring distinct outer edges (Fig. 5b). POV beams with complete polygonal structure emerge only when all three modes are phase-locked simultaneously, as shown in Fig. 5c.

## Discussion

Currently, based on the longitudinal mode-locked oscillator, we have realized FPOV pulses with square, pentagonal and hexagonal patterns. The FPOVs are composed of three different LG modes, fundamentally governed by the frequency and phase-locking between these modes. This property demands more precise control compared to the single high-order HG and LG vortex mode generation, as maintaining phase coherence among multiple transverse modes introduces additional complexity in cavity stabilization. However, this multi-mode nature may provide enhanced stability over single LG modes, which are more susceptible to environmental noise during free-space propagation or in complex media. Previous studies suggest that such mode combinations can exhibit superior propagation resilience in structured or turbulent media^44,45^. Moreover, generating FPOVs imposes more stringent requirements on the laser configuration and mode-locking condition, which limit the tuning range of achievable FPOV modes. This makes their tunability more challenging compared to single high-order LG vortex mode generation. Furthermore, unlike high-order LG modes that contain a single phase singularity, FPOVs exhibit multiple singularities at their vertices. This unique structural feature provides greater flexibility for applications in optical tweezers, material processing, and quantum information technology, where precisely tailored phase distributions and structured optical fields offer distinct advantages.

According to the tendency shown in Fig. 2, decreasing the distance *L* will enable the generation of triangular FPOV and increasing *L* could in turn, generate femtosecond polygons with more than six corners. Principally, it can enable smooth and repeatable switching between different polygonal vortex beams. In the experiment, we have successfully demonstrated the generation of heptagonal and octagonal POV beams in CW regime (see Fig. S4, Supplementary Information). However, stable longitudinal mode locking has not yet been achieved, as evidenced by either a CW spike in the spectrum or a Q-switched envelope in the pulse train. This is likely arising from their higher mode order, which results in insufficient power density to sustain stable longitudinal mode locking via SESAM. The generation of POVs with higher-order symmetries (more than eight corners) has not been realized yet. This is most likely caused by the constrained adjustable range of the cavity stability region and the drastic mode mismatch between the laser and the pump beam. Achieving higher-order modes requires further adjustments of the distance *L*, which will alter the cavity stability condition and push the laser cavity closer to the stability boundary. Under such conditions, laser oscillation becomes less favorable, and achieving optimal higher-order modes becomes increasingly more challenging due to heightened sensitivity to positional adjustments. Additionally, the change of distance *L* will also affect the fundamental mode beam size and aggravate the mode mismatch. To overcome these limitations, we could optimize the laser cavity design with an expanded stability range and a reduced sensitivity of the beam size to the SESAM position variation. Together with an increased pump power and the hybrid pumping scheme integrating off-axis and non-collinear pumping techniques^18^, the generation of tunable higher-order FPOV pulses can be enabled. Besides, the current pulse duration of FPOV beams is limited by both the dispersion management and the gain bandwidth of the laser crystal, which is expected to be further shortened to ~100 fs by replacing the GTI mirror with broadband dispersion mirrors as well as using gain media with broadband emission spectrum.

In conclusion, we have demonstrated the generation of QFD-HG pulses from a SESAM mode-locked Yb:KGW solid-state laser oscillator by employing a translation-based off-axis pumping scheme. Using an AMC, the QFD-HG pulses were converted to the FPOV pulses with a polygonal light intensity distribution. By changing the cavity length and altering the laser cavity, FPOV pulses with different intensity structures can be generated with square, pentagonal and hexagonal patterns. This is the first time FPOV beams have been generated. All the FPOV pulses have average powers beyond one watt and pulse durations of less than 500 fs. Furthermore, by analyzing the interference patterns of three kinds of FPOV pulses, we verified the phase singularity and calculated the topological charge number, which exhibited excellent agreement with simulations. The reported FPOV source can generate different light intensity structures and introduces a new degree of freedom to femtosecond vortex beams. It provides a platform for the development of new material processing techniques and femtosecond optical tweezers.

## Materials and methods

### Frequency-degenerate HG mode

When a regular laser cavity operates freely and outputs a single transverse mode, the output mode will satisfy the Helmholtz equation, and the separable eigenmodes in the Cartesian coordinate system can be given by the HG mode:

1 $${\psi }_{n,m,s}^{\text{HG}}(x,y,z)=\sqrt{\frac{2}{L}}\,{S}_{n,m}^{({HG})}(x,y,z)\exp [i{k}_{n,m,s}\widetilde{z}-i(m+n+1){\theta }_{G}(z)]$$

Here, the light intensity distribution of HG mode can be given

2 $${S}_{n,m}^{\text{HG}}(x,y,z)=\frac{1}{\sqrt{{2}^{m+n-1}\pi m!n!}}\frac{1}{\omega (z)}{H}_{n}\left[\frac{\sqrt{2}x}{\omega (z)}\right]{H}_{m}\left[\frac{\sqrt{2}x}{\omega (z)}\right]\exp \left[-\frac{{x}^{2}+{y}^{2}}{\omega {(z)}^{2}}\right]$$

where *L* is the cavity length, *H*_*n*_(•) represents the Hermite polynomials of *n*-th order, *θ*_*G*_(*z*) = tan^−1^(*z/z*_*R*_) is the Gouy phase, *k*_*n,m,s*_ = 2π*f*_*n,m,s*_*/*c, *f*_*n,m,s*_ is the eigenmode frequency, *c* is the speed of light, $\widetilde{z}=z+({x}^{2}+{y}^{2})z/[2({z}^{2}+{z}_{R}^{2})]$, $\omega (z)={\omega }_{0}\sqrt{1+(z/{z}_{R}^{2})}$, ${\omega }_{0}=\sqrt{(\lambda {z}_{R})/\pi }$ is the beam radius at the waist, and *λ* is the emission wavelength. The eigenmode frequency *f*_*n,m,s*_ can be described as ${f}_{n,m,s}=s\varDelta {f}_{L}+(n+m+1)\varDelta {f}_{T}$, where $\varDelta {f}_{L}$ is the longitudinal mode spacing and $\varDelta {f}_{T}$ is the transverse mode spacing, *s* is the longitudinal mode indices, *m* and *n* are the transverse mode indices.

Compared with the regular laser cavity, if the cavity meets the reentrant condition of two-dimensional coupled harmonic oscillators in SU (2) Lie algebra, it could be called frequency-degenerate cavity (FDC) and the generated laser is called FD-HG mode. For a FDC, the cavity configuration will be a FD state and satisfy the conditions of mode-spacing ratio $\varOmega =\varDelta {f}_{T}/\varDelta {f}_{L}=P/Q\in Q$, where *P* and *Q* are coprime integers, Q represents the rational number field. The longitudinal mode spacing ∆*f*_*L*_ and the transverse mode spacing ∆*f*_*T*_ can be respectively given by ref. ^46^

3 $$\varDelta {f}_{L}=c/2L$$

4 $$\varDelta {f}_{T}==\frac{\varDelta {f}_{L}}{\pi }\,{arccos}\, {({AD})}^{\frac{1}{2}}$$

There *A* and *D* are the elements in $$\left(\begin{array}{cc}A & B\\ C & D\end{array}\right)$$ which is the one-way transfer matrix between two end mirrors. And the mode-spacing ratio can be simplified as

5 $$\varOmega =\varDelta {f}_{T}/\varDelta {f}_{L}=\frac{{arccos}\, {({AD})}^{\frac{1}{2}}}{\pi }$$

By changing the one-way transfer matrix of the laser cavity, the mode-spacing ratio $\Omega$ can become a rational number, and the FD-HG mode will be generated from the cavity, which is given by

6 $${\varPsi }_{{n}_{0},M,{\phi }_{0},\varOmega }^{\text{FD}-\text{HG}}(x,y,z)=\frac{1}{{2}^{M/2}}{\sum }_{K=0}^{M}\sqrt{\frac{M!}{K!(M-K)!}}\,{e}^{{iK}{\phi }_{0}}{\psi }_{{n}_{0}+{QK},0,{s}_{0}-PK}^{(\text{HG})}(x,y,z)$$

Here, *n*_*0*_ represents the minimum transverse order, and *s*_*0*_ represents the maximum longitudinal order. The FD mode was composed of many HG modes, which are part of the FD family. *M* + 1 stands for the number of HG modes in the FD family. The phase factor *ϕ*_0_ represents the phase difference between different modes and exhibits different rays of the geometric periodic orbits^47^. Considering that the standing-wave mode inside the laser resonant cavity is composed of the superposition of positive and negative oscillations, the phase state expression is ${e}^{{iK}{\phi }_{0}}+{e}^{-{iK}{\phi }_{0}}=\cos (K{\phi }_{0})$. The outputted FD-HG mode consists of many HG modes with the transverse mode-locking belonging to an FD family. It allows the HG modes of an FD family to have the same eigenmode frequency in an FD laser. It suggests that the longitudinal mode locking and transverse mode locking can be achieved simultaneously for the FD-HG modes without additional beat frequency signals. And it applies to the combinations of any modes in the FD family.

### Polygonal optical vortex

The FD-HG modes are composed of multiple HG modes in the FD family. But since a laser is not monochromatic and has a spectral linewidth, when the laser cavity is adjusted to gradually move away from the FD state, the oscillating mode does not abruptly degrade from the FD-HG mode to the non-frequency-degeneracy mode of a single HG mode. Under these conditions, the generated laser oscillating mode is called the QFD-HG mode and is usually formed by the phase locking of several HG modes in the FD family. Compared to FD-HG mode, it has very few mode components but still has the same eigenmode frequency and longitudinal mode frequency comb teeth. This indicates that both transverse and longitudinal mode locking can still be achieved simultaneously in QFD-HG mode, resulting in stable and clean pulse trains.

For the POV, it can be converted by a QFD-HG mode, which is the combination of three HG modes in the FD family. And the QFD-HG mode can be given by

7 $${\varPsi }_{N,\phi ,\varOmega }^{{\text{QFD}}-{\text{HG}}}={c}_{0}({\psi }_{N-Q,0,S+P}^{{\text{HG}}}{\exp} (-i\phi ) -\,{\psi }_{N+Q,0,S-P}^{{\text{HG}}}\exp ( i\phi))+{\psi }_{N,0,S}^{{\text{HG}}}$$

Here, ${c}_{0}$ represents modulation coefficient, the phase factor *ϕ* represents the phase difference between the three HG modes including HG_*N-Q*,0_, HG_*N*,0_ and HG_*N+Q*,0_ modes in the FD family.

With the mode converter, the POV can be generated and described as the combination of three corresponding LG modes with their phase-locking:

8 $${\varPsi }_{N,\phi ,\varOmega }^{\text{POV}}={c}_{0}({\psi }_{0,N-Q,S+P}^{\text{LG}}\exp (-i\phi )-{\psi }_{0,N+Q,S-P}^{\text{LG}}\exp (i\phi ))+{\psi }_{0,N,S}^{\text{LG}}$$

with the LG mode function:

9 $${\psi }_{p,l,s}^{\text{LG}}(r,\varphi ,z)=\sqrt{\frac{2p!}{\pi \left(p+\left|l\right|\right)!}}\frac{1}{\omega \left(z\right)}{\left[\frac{\sqrt{2}r}{\omega \left(z\right)}\right]}^{\left|l\right|}{L}_{p}^{\left|l\right|}\left[\frac{2{r}^{2}}{{\omega }^{2}\left(z\right)}\right]\exp \left[-\frac{{r}^{2}}{{\omega }^{2}\left(z\right)}\right]\exp ({il}\varphi )\times \exp [i{k}_{n,m,s}\widetilde{z}-i(m+n+1){\theta }_{G}(z)]$$

where (*r*, *φ*, *z*) represents the cylindrical coordinate, ${L}_{p}^{{|l|}}$ and represents the associated Laguerre polynomials with radial and azimuthal indices of *p* and *l*. The POV consists of three LG modes, including LG_0,*N-Q*_ LG_0,*N*_, and LG_0,*N+Q*_ modes and has the light intensity distribution of a *Q*-gon-shaped route in the beam pattern.

### Conversion from the HG mode to the LG mode with an AMC

In the experiment, the QFD-HG mode was converted into the POV beam by an AMC system. The principle of mode conversion by an AMC has been described in ref. ^37^. The input HG mode can be decomposed into a set of HG modes of the same order:

10 $${u}_{{nm}}^{{\rm{HG}}}\left(\frac{x+y}{\sqrt{2}},\,\frac{x-y}{\sqrt{2}},\,z\right)={\sum }_{k=0}^{N}\,b\left(n,\,m,\,k\right){u}_{N-k,k}^{{HG}}(x,\,y,\,z)$$

where *N* = *n* + *m*, and the coefficient is

11 $$b(n,m,k)={\left(\frac{(N-k)!k!}{{2}^{N}n!m!}\right)}^{1/2}\times \frac{1}{k!}\frac{{d}^{k}}{d{t}^{k}}[{(1-t)}^{n}{(1+t)}^{m}{]}_{t=0}$$

After passing the AMC, a relative phase difference of π/2 is introduced between the successive components. The LG mode can be composed by combining these different components, which can be described as

12 $$\mathop{\sum }\limits_{k=0}^{N}\,{i}^{k}b(n,\,m,\,k)\,{u}_{N-k}^{{\rm{HG}}}(x,\,y,\,z)={u}_{{pl}}^{{\rm{LG}}}(x,\,y,\,z)$$

where the index *p* is the minimum of *n* and *m* (min (*n*, *m*)), and the index *l* is the absolute value of *n–m* (|*n–m*|). In this way, an HG mode can be converted into an LG mode by an AMC.

The simulated transition process from QFD-HG mode to POVs is shown in Fig. S1 (Supplementary Information).

## Supplementary information

Supplementary Information for "Generation of femtosecond polygonal optical vortices from a mode-locked quasi-frequency-degenerate laser"

## Data Availability

All data are available from the corresponding authors upon reasonable request.
