# Supplementary material for: Generation of femtosecond polygonal optical vortices from a mode-locked quasi-frequency-degenerate laser
Source: Light Sci Appl. 2025 Jun 23;14:222. doi: 10.1038/s41377-025-01902-1 (PMC12183311; doi:10.1038/s41377-025-01902-1)
Supplement: Supplementary file 1 — Supplementary Information for "Generation of femtosecond polygonal optical vortices from a mode-locked quasi-frequency-degenerate laser" [file 41377_2025_1902_MOESM1_ESM.docx]

**Supplementary Information for**

**Generation of femtosecond polygonal optical vortices from a mode-locked quasi-frequency-degenerate laser**

Hongyu Liu^1, †^, Lisong Yan^1, †^, Liang Wang^1, †^, Dongfang Li^1^, Shenao Zhang^1^, Xin Liu^1^, Heyan Liu^1^, Kunjian Dai^1^, Qing Wang^2^, and Jinwei Zhang^1, *^

1, School of Optical and Electronic Information and Wuhan National Laboratory for Optoelectronics, Huazhong University of Science and Technology, Wuhan 430074, China

2, School of Optics and Photonics, Beijing Institute of Technology, Beijing 100081, China

^†^These authors contributed equally to this work.

^*^Corresponding author: jinweizhang@hust.edu.cn


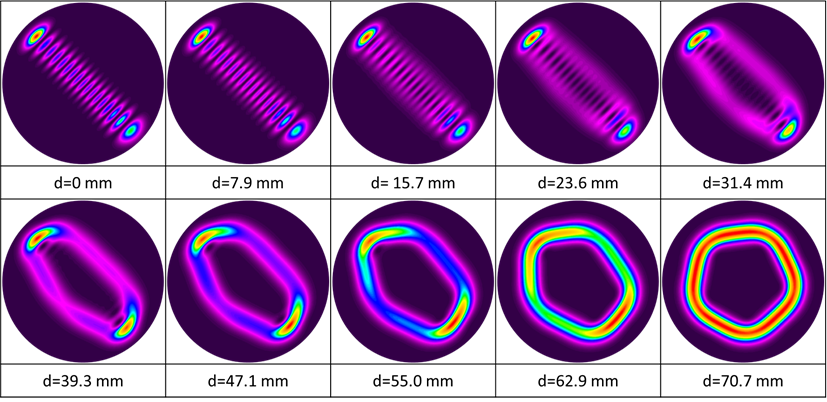


Fig. S1. The simulation of the transition from QFD-HG mode to pentagonal POV beam by an AMC. Here, d represents the propagation distance after passing through the first cylindrical mirror. When the beam passes through the second cylindrical mirror with d=$\sqrt{2}f$ (*f* is the focal length of the two cylindrical mirrors and is assumed to be 50 mm here), the mode conversion of the AMC is optimal.


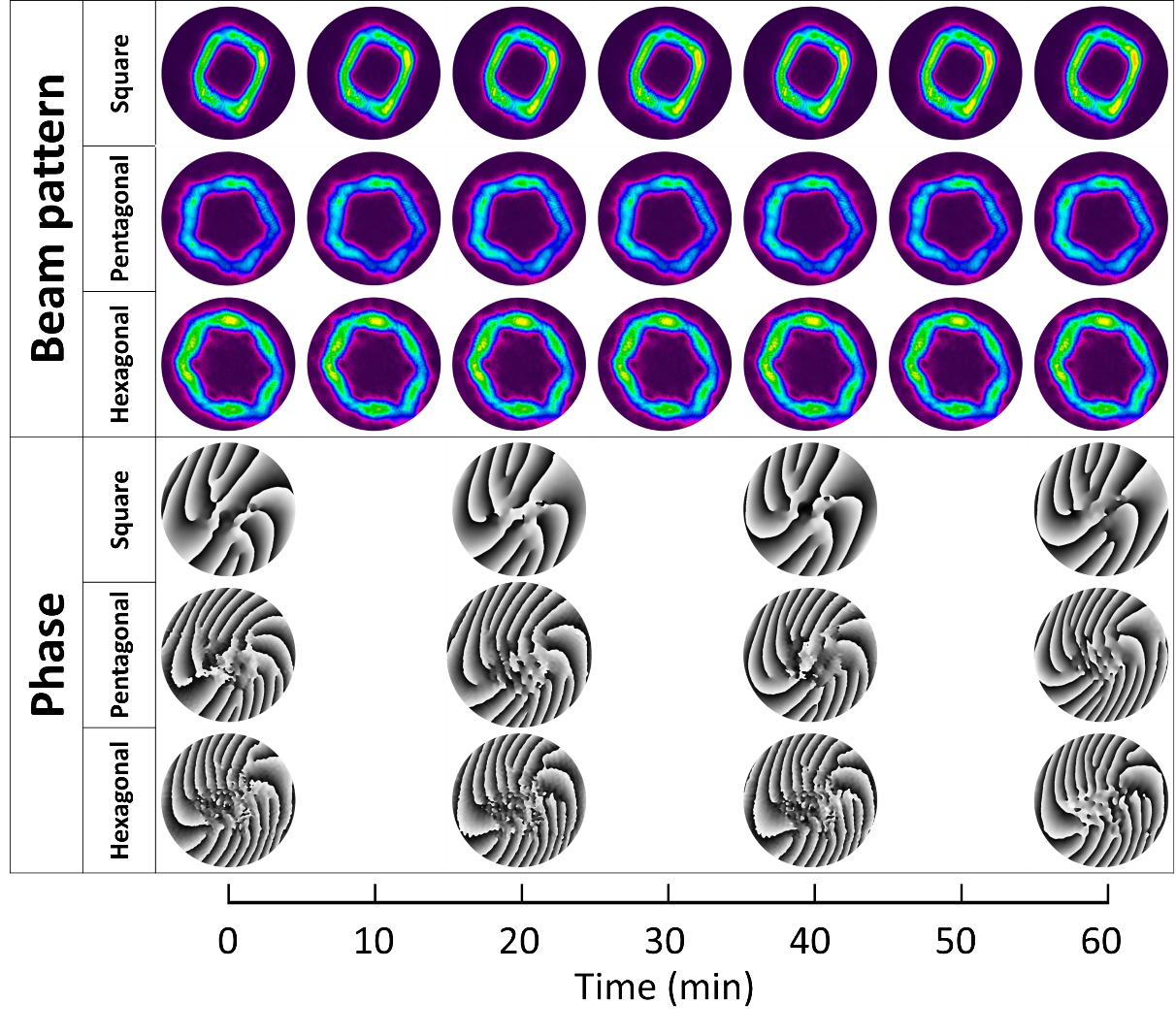


Fig. S2. The measured beam profiles (at ten-minute intervals) and the recovered phase maps (at twenty-minute intervals) for square, pentagonal and hexagonal FPOV pulses within one hour.


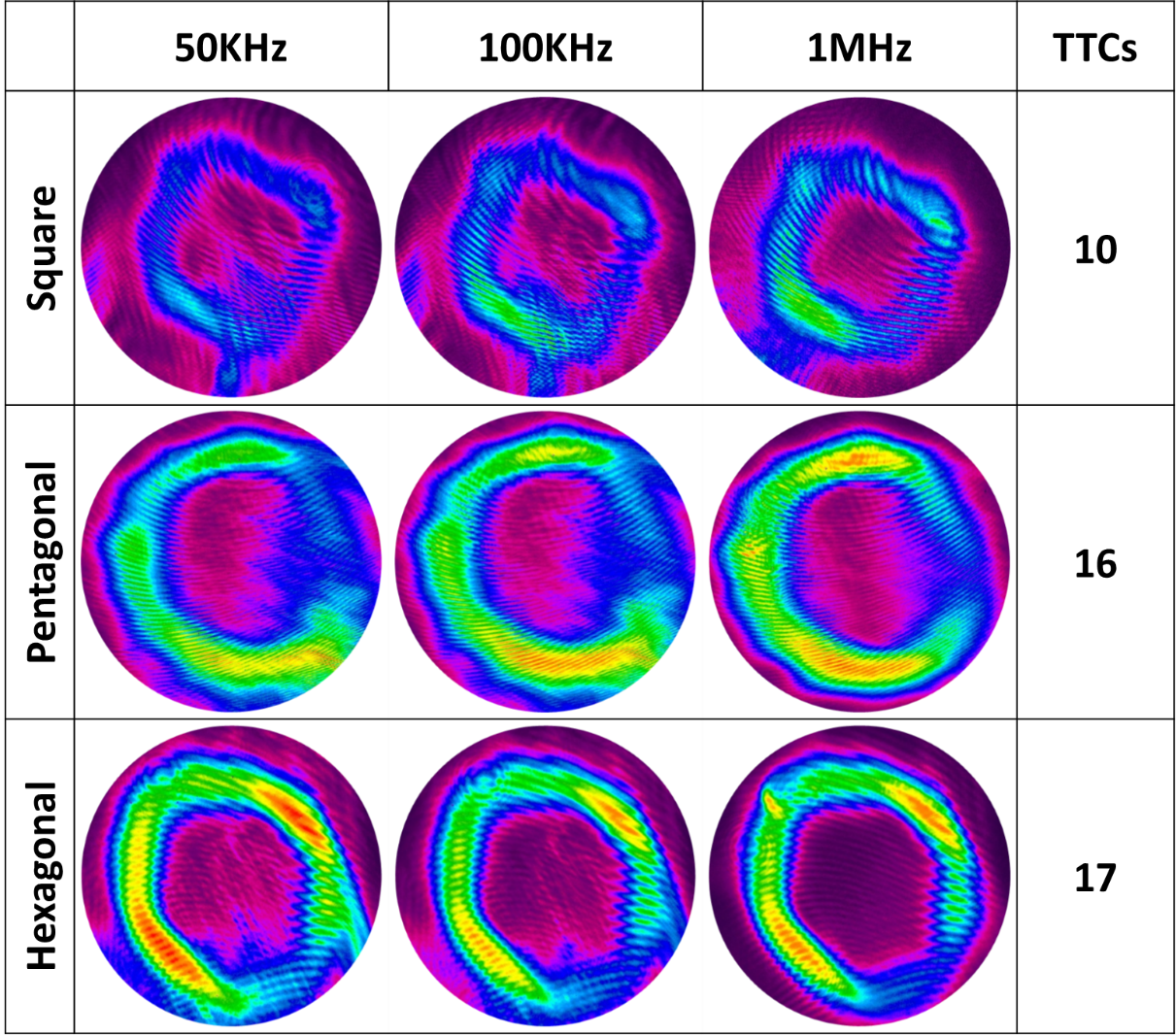


Fig. S3. The measured interference patterns of square, pentagonal and hexagonal FPOV pulses with pulses selection and repetition frequency reduction by using a high-speed Pockels cells. The repetition frequency was reduced to 50 kHz, 100 kHz, and 1 MHz, respectively. At three different repetition frequencies, the three FPOVs show stable total topological charges (TTCs) of 10, 16 and 17, respectively.


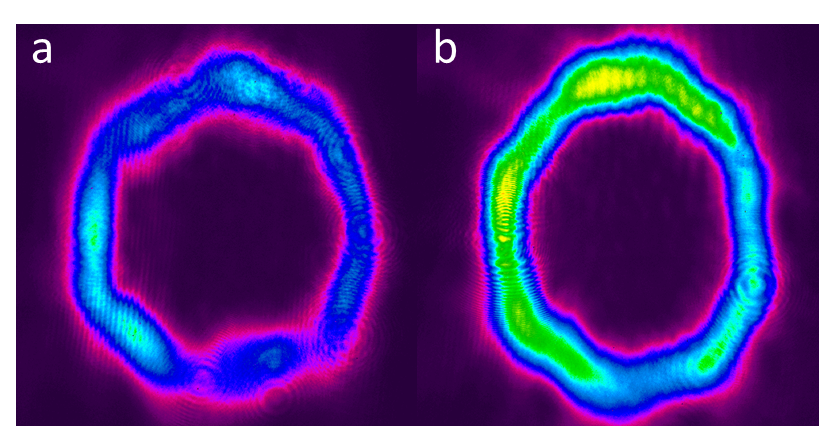


Fig. S4. The measured beam patterns of heptagonal (a) and octagonal (b) POVs operated in continuous-wave regime.
